# Effectiveness of healthcare educational and behavioral interventions to improve gout outcomes: a systematic review and meta-analysis

**DOI:** 10.1177/1759720X18807117

**Published:** 2018-11-19

**Authors:** Karishma Ramsubeik, Laurie Ann Ramrattan, Gurjit S. Kaeley, Jasvinder A. Singh

**Affiliations:** Division of Rheumatology, University of Florida College of Medicine, Jacksonville, FL, USA; Division of Rheumatology, University of Florida College of Medicine, Jacksonville, FL, USA; Division of Rheumatology, University of Florida College of Medicine, Jacksonville, FL, USA; Division of Clinical Immunology and Rheumatology, University of Alabama, Birmingham VA Medical Center, Faculty Office Tower 805B, 510 20th Street South, Birmingham, AL, 35294, USA

**Keywords:** behavioral intervention, educational intervention, gout, health behavior, health education, health personnel, hyperuricemia, outcomes, practice, self-management

## Abstract

**Background:**

We aimed to systematically review the effectiveness of healthcare behavioral and education interventions for gout patients on clinical outcomes.

**Methods:**

We searched multiple databases to identify trials or observational studies of educational or behavioral interventions in gout. Risk of bias was assessed with the Cochrane tool for randomized control trials (RCTs) and the Newcastle–Ottawa Scale for observational studies. We estimated odds ratios (ORs) for categorical and standardized mean difference (SMD) for continuous measures using a random-effects model.

**Results:**

Overall, eight (five RCTs and three observational) studies met the inclusion criteria and examined pharmacist-led interventions (*n* = 3), nurse-led interventions (*n* = 3) and primary care provider interventions (*n* = 2). Compared with the control intervention (usual care in most cases), a higher proportion of those in the educational/behavioral intervention arm achieved serum urate (SU) levels <6 mg/dl, 47.2% *versus* 23.8%, the OR was 4.86 [95% confidence interval (CI), 1.48, 15.97; 4 RCTs] with moderate quality evidence. Compared with the control intervention, a higher proportion of those in the educational/behavioral intervention arm were adherent to allopurinol, achieved at least a 2 mg/dl decrease in SU, achieved an SU < 5 mg/dl, had a reduction in the presence of tophi at 2 years, had improved quality of life as assessed with SF-36 physical component scores, had a higher knowledge about gout and higher patient satisfaction (moderate-low quality evidence).

**Conclusion:**

Educational and behavioral interventions can improve gout outcomes in the short-intermediate term. Randomized trials are needed to assess its impact on long-term gout outcomes.

## Introduction

Gout is a chronic disease resulting from the deposition of urate crystals and the associated activation of the innate immune system, leading to systemic inflammation. The crystals may be deposited in joints or soft tissue leading to an acute inflammatory response characterized by painful episodes. The prevalence of gout is increasing and represents a significant burden in terms of both direct healthcare costs and health-related quality of life outcomes.^1^ The American College of Rheumatology (ACR) formulated a treatment guideline for the management of gout in 2012.^2^ However, despite the existence of effective therapies and the development of evidence-based guidelines, there are still significant practice variations and gaps between recommended care and the current practice.^3^ The difficulties in gout management are multiple including poor patient–physician communication, disease and treatment misperceptions, and low/suboptimal adherence to treatments for gout, which lead to active disease and an inability to achieve target serum urate (SU) levels, an important treatment goal according to the ACR gout treatment guideline,^2^ which has been linked to improved patient outcomes. These treatment gaps include lack of education, financial resources and self-motivation to take the medication.^4^

Achieving behavior change is complex and requires the expertise and competencies of both patients and healthcare professionals. Changing knowledge, attitude, beliefs, and associated behavior is key to medication adherence interventions and improved disease self-management.^5,6^ Improving medication adherence encompasses frameworks, which includes attempting to enhance intention and knowledge through education, which can take various forms. These may include verbal, written material or mobile health material, change attitude and intensify motivation through counseling and improve associated behavior through cues, reminders and self-monitoring.^5–9^ Education and counseling are the most frequently studied measures.^5,6^

Information about medication indications, frequency, dose, side effects, and the importance of medications for illness management are critical components of targeted educational/behavioral interventions. Counseling aims to change negative thoughts about medications and increase motivation and often involves patient contact by a healthcare provider such as a pharmacist, nurse or physician.^5^ Even when patients recognize the value of their medications, some still have difficulty adhering to treatment regimens. Research has demonstrated that electronic reminders and cues can effectively improve adherence.^8,9^ Self-management programs improve health outcomes.^10^ Recently, several gout-specific patient interventions have been studied, including nurse- and pharmacist-led programs. A multi-stakeholder medication consensus conference organized by the Agency for Healthcare Research and Quality identified novel aspects of medication adherence and self-management strategies with patient-centeredness as the main theme.^11,12^

The effectiveness of behavioral or educational/behavioral intervention programs for adults with gout has yet to be systematically and comprehensively assessed. Thus, our objective was to evaluate available evidence for the effect of educational or behavioral healthcare interventions on clinical and patient-reported outcomes in patients with gout.

## Methods

This review was reported according to the Preferred Reporting Items for Systematic Reviews and Meta-Analyses (PRISMA) statement.^13^ The protocol was registered in the Prospero International Prospective Register of Systematic Reviews (registration number CRD42018106245).

## Data sources and searches

We considered any randomized controlled trial (RCT), controlled clinical trial, open-label trial, and observational study. We included patients at least 18 years of age with gout who either met the preliminary 1977 ACR criteria for acute arthritis of primary gout,^14^ the 2015 ACR-European League Against Rheumatism (EULAR) gout classification criteria^15^ or had a clinical diagnosis of gout. We considered both full text published studies, as well as abstracts, as long as at least one outcome of interest was reported in the abstract. The following electronic databases were searched: *PubMed, Embase, CINAHL, PsycINFO* and *Scopus* from the start date of the database to April 2018. We also searched *Clinicaltrials.gov* and the National Information Center on Health Services Research and Healthcare Technology (NICHSR) for unpublished trials and studies.

Search keywords were developed with the assistance of a research librarian (KHS) and included ‘health education’, ‘behavior control’, ‘information dissemination’, ‘access to information’, ‘patient compliance’, ‘self-management’, ‘educational models’, ‘choice behavior’, ‘telemedicine’, ‘social media’, ‘health knowledge, attitudes, practice’, ‘health behavior’, ‘needs assessment’, ‘patient participation’, ‘health personnel’, ‘gout’, and ‘hyperuricemia’. Whenever possible MeSH terms and advanced searched strategies were used. The electronic database searches were complemented by manually reviewing the references of relevant reviews and included studies.

Studies were included in the review if the underlying diagnosis was gout, there was a behavioral or educational/behavioral intervention targeting patient, provider or systems factors related to gout care, data on one or more outcome measures was reported and it was an original study published in a peer-reviewed journal, or a published abstract.

Outcome measures included the lowering of SU, achieving a target SU (<6 or <5 mg/dl), reduction of gout flares, presence of tophi, reduction in the number and size of tophi, treatment adherence to medications for gout, physical function, quality of life, patient satisfaction, patient knowledge, attitudes and behavior, patient–physician communication, trust in physicians, shared decision-making, healthcare utilization and healthcare costs.

## Study selection and data extraction

Two abstractors (KR and LAR) independently assessed all titles and abstracts. We used EndNote X7 software (Clarivate Analytics, Philadelphia, PA, USA) to manage the records retrieved from electronic database searches. For all potentially eligible studies, we obtained the full text papers and assessed their eligibility. Two independent abstractors (KR and LAR) captured all pertinent data from each eligible study directly into a customized data extraction form created in Microsoft Excel.

We extracted the following characteristics from all included studies: study sample demographics (age, sex, race), literacy level, socioeconomic status, follow-up time, clinical outcomes [SU and number/frequency of gouty flares, adherence to urate-lowering therapy (ULT) and other therapies, presence of tophi, reduction in number and size of tophi] patient-reported outcomes (quality of life, function, patient satisfaction), patient-relevant outcomes (patient knowledge, attitudes and behavior, patient–physician communication, trust in physicians, shared decision-making) and health services outcomes (healthcare utilization and costs). We analyzed observational studies separately from the RCTs. Any disagreements between the two reviewers were resolved by a discussion or in consultation with an arbiter (JAS). Any disagreements were discussed until consensus was reached.

## Risk of bias (quality) assessment

The risk of bias in RCTs was assessed using the Cochrane risk of bias tool independently by two reviewers (KR and LAR),^16^ and consensus was achieved by discussion or by the help of an arbiter (JAS). The domains assessed included adequacy of sequence generation, allocation concealment, blinding of participants and personnel, blinding of outcome assessments, incomplete outcome data addressed and free of selecting reporting.

Observational study quality was assessed using the Newcastle–Ottawa scale.^17^ This is a risk of bias tool designed for quality assessment of observational studies with separate scales for case-control and cohort studies. It assigns up to a maximum of nine points for the least risk of bias in three domains: (1) selection of study groups (four points); (2) comparability of groups (two points); and (3) ascertainment of exposure and outcomes (three points) for case-control and cohort studies, respectively. The score can range 0–9, with nine representing the best quality score.

We evaluated the certainty of evidence for each outcome by using the Grading of Recommendations, Assessment, Development, and Evaluation (GRADE) approach and resolved any discrepancies.^18^ All GRADE domains, that is, risk of bias, inconsistency, imprecision, indirectness, publication bias for RCTs (which start at high quality and can be downgraded for these criteria), and large effect, presence of a dose response, and plausible opposing confounders for observational studies (which start at low quality and can be upgraded for these criteria) were assessed. According to the GRADE, the certainty of evidence was presented as high, moderate, low, or very low.^19^

## Strategy for data synthesis

All analyses were performed using RevMan 5.3.^20^ We calculated the odds ratio (OR) with 95% confidence intervals (CIs) for categorical measures and standardized mean difference (SMD) for continuous measures. We performed the meta-analyses, where feasible. Sensitivity analyses was also performed to test for robustness of the results and to explain any heterogeneity.

## Results

**Figure 1.**
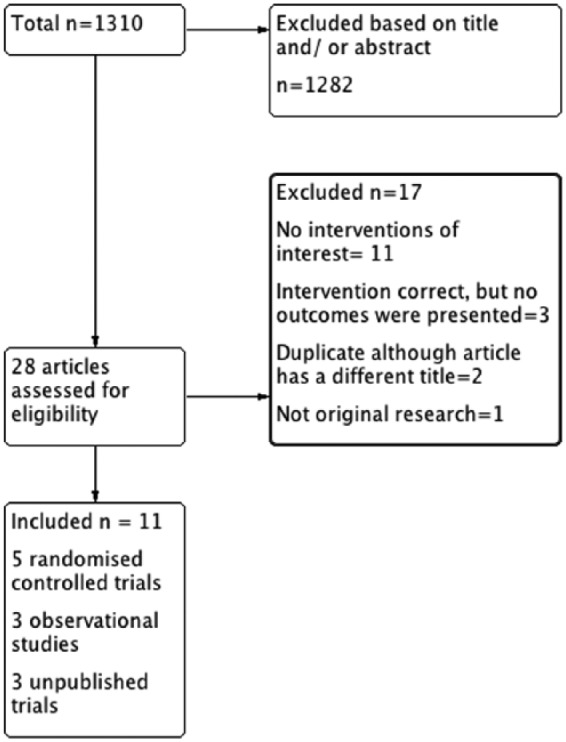
Study selection flow chart that shows included studies and the results for exclusion of studies.

The search resulted in 1310 potentially relevant titles and abstracts (Figure 1). A total of 28 articles qualified for the full text review, of which 12 met inclusion criteria. Of these, five were RCTs (three abstracts), three were observational cohort studies (two abstracts) and three were unpublished, with two underway and one completed in 2015 but not published (Table 1). Enough data were available in the abstracts for their inclusion in the analysis.

**Table 1.** Characteristics of eligible gray literature studies, or those pending publication due to ongoing recruitment or recent completion.

Database	Study ID/ sponsor		Number of patients	Intervention arms	Current status	Year of completion
	Record source/ award ID	UI				
NICHSR	RePorter/K23AR053856	20113190	Not reported	Educational and telephone counseling	Completed	2012
Clinicaltrials.gov	NCT02741700		300	Narrative/ storytelling	Recruiting	2020
Clinicaltrials.gov	NCT02790463*		1250	Behavioral: pharmacist-led intervention automated telephone IVR and direct telephone contact	Active, not recruiting	2018

* Same as the study reported as Mikuls and colleagues^26^ as an abstract.

IVR, interactive voice recognition; NCT, ClinicalTrials.gov identifier; UI, unique identifier.

Study characteristics are listed in Table 2. The interventions included pharmacist-led educational and management interventions^21–23^ (*n* = 3), nurse-led educational and management interventions^24–26^ (*n* = 3), and an educational/behavioral intervention^27^ or a behavioral intervention^28^ targeting primary care providers (*n* = 2).

**Table 2.** Characteristics of included studies.

	Studies							
	Doherty and colleagues^24^	Whiteman and colleagues^21^	Goldfien and colleagues^22^	Bulbin and colleagues^27^	Mikuls and colleagues^23^	Rees and colleagues^26^	Leyva and colleagues^28^	Yoo and colleagues^25^
**Number of patients**	517	52	77	819	1412	106	13	100
Single *versus* multicenter	Multicenter	Single	Single	Multicenter	Single	Multicenter	Single	Single
Average age control group (years; SD)	64.0 (SD not reported)	Not reported	58.0 (2.0)	67 (13.6)	Not reported	61.0 (11)	59 ± 8.9 years	Not reported
Average age intervention group (years; SD)	62.0 (SD) not reported	Not reported	60.9 (2.0)	66.8 (16.1)	Not reported	61.0 (11)	59 ± 8.9 years	Not reported
White (%)	Not reported	Not reported	30	95	45	Not reported	39	Not reported
Female (%)	10.5	Not reported	12	26	Not reported	6	Not reported	Not reported
Follow-up duration (months)	24	23	6.5	6	24	12	3	3
Decrease in SU intervention group mg/dl	1.4	2.86	1.5	Not reported	Not reported	1.09	2.4	0.64
Health person delivering education	Nurse	Pharmacist	Pharmacist	Physician	Pharmacist	Nurse	Physician	Nurse
ULT	Allopurinol	Allopurinol Febuxostat	Allopurinol Probenecid Febuxostat	Allopurinol Febuxostat	Not reported	Allopurinol	Allopurinol	Not reported
Intervention	Addressing illness perceptions and involved patients in management decisions	Giving information about gout, its treatment, dietary, lifestyle modification and the importance of compliance with ULT. Pharmacist performed ongoing clinical review and monitoring/adjustment of treatment.	Providing written educational material on gout at program entry Pharmacists allowed to order labs and change orders for medication.	Engagement of intervention site staff, surveys of provider performance improvement preferences, and onsite live and enduring online education. Electronic health record reminders	Allopurinol prescribing by pharmacist Patient outreach conducted primarily *via* telephone IVR system	Delivering education, individualized lifestyle advice and appropriate ULT Followed up by telephone or in person to monitor clinical progress and success of lifestyle modification, and titrate ULT	Developing and implementing a personalized health plan Initial interview focused on formulating a goal. Patient selected goal indicating their starting and desired status using a numerical scale	Delivering face-to-face gout education including an information leaflet about lifestyle advice and ULT

IVR, interactive voice recognition; SD, standard deviation; SU, serum urate; ULT, urate-lowering therapy.

Of the five randomized trials, two involved pharmacist-led interventions,^22,23^ two involved nurse-led interventions^24,25^ and one involved a primary care provider intervention.^27^ Table 2 shows details of the interventions in the published trials. The pharmacist-led interventions consisted of (1) a pharmacist-staffed gout telephone management program where the clinical pharmacist was authorized to order relevant laboratory tests and to initiate or to change orders for the ULT medications and flare prophylaxis medications^22^ and (2) a pharmacist-driven intervention including patient outreach *via* a telephone interactive voice recognition (IVR) system to assess adherence, encourage SU monitoring, provide patient-focused gout education and adjust allopurinol dosage.^23^ The nurse-led interventions included (1) face-to-face education by a specialist nurse who also provided an information leaflet about lifestyle advice and ULT^25^ and (2) nurse-led care by nurses trained about gout and its management according to recommended best practice (EULAR and British Society of Rheumatology guidelines) involving full information, addressing illness perceptions, and involving patients in management decisions.^24^ The primary care provider’s intervention consisted of engagement of intervention site staff, surveys of provider performance improvement preferences and onsite live and enduring online education.^27^

The RCT outcomes included achieving a goal SU < 6 mg/dl,^22–24,27^ SU < 5 mg/dl,^24^ presence of tophi at 2 years,^24^ allopurinol treatment adherence at 1 year,^23^ being monitored with SU at 6 months,^27^ achieving at least a 2 mg/dl decrease in SU at week 26,^22^ taking ULT at the end of the study period^24,27^ and the likelihood of being monitored at 6 months,^27^ patient satisfaction based on a visual analogue scale and patient satisfaction questionnaire, patient’s knowledge about gout,^25^ proportion of days covered at 1 year,^23^ change in SU,^22,23^ mean gout flare frequency,^24^ SF-36 norm-based physical component scores,^24^ SU at 2–3 months,^25^ drug compliance at 2–3 months^25^ and ending allopurinol dose^23,24^ (Table 3).

**Table 3.** Summary of the effectiveness of educational and behavioral interventions by outcomes with associated GRADE^18^ ratings.

Outcome or subgroup	# Studies/participants	Treatment arms in the included studies	Outcome: intervention versus control n/N (%)	Effect estimate odds ratio (M–H, random, 95% CI); heterogeneity I2%	GRADE rating
SU-lowering					
SU < 5 mg/dl	1/517	Nurse-led education *versus* general practitioner care^24^	224/255 (87.8%) *versus* 42/262 (16%)	37.85 [22.96, 62.40]; N/A	Moderate^1^
SU < 6 mg/dl	4/2825	Pharmacist-led education and management *versus* usual care^22^ Primary care provider education *versus* usual care^27^ Pharmacist- led education and management *versus* usual care^23^ Nurse-led education *versus* general practitioner care^24^	644/1,365 (47.2%) *versus* 347/1460 (23.8%)	4.86 [1.48, 15.97]; 97%	Moderate^1^
Achieving at least a 2 mg/dl decrease in SU at week 26	1/77	Pharmacist-led education and management *versus* usual care^22^	14/37 (37.8%) *versus* 5/40 (12.5%)	4.26 [1.35, 13.44]; N/A	Low^2^
ULT adherence and SU monitoring outcomes					
ULT adherent: PDC ⩾ 0.8 at 1 year	1/1412	Pharmacist-led education and management *versus* usual care^23^	300/630 (47.6%) *versus* 277/782 (35.4%)	1.66 [1.34, 2.05]; N/A	Moderate^1^
Being monitored with SU at 6 months	1/819	Primary care provider education *versus* usual care^27^	351/443 (79.2%) *versus* 201/376 (53.5%)	3.32 [2.45, 4.51]; N/A	Moderate^1^
Patients taking ULT at 6 or 24 months^*^					
6 months	1/819	Primary care provider education *versus* usual care^27^	271/443 (61.2%) *versus* 201/376 (53.5%)	1.37 [1.04, 1.81]; N/A	Moderate^1^
24 months	1/517	Nurse-led education *versus* general practitioner care^24^	247/255 (96.9%) *versus* 141/262 (53.8%)	26.50 [12.58, 55.80]; N/A	Moderate^1^
Tophi					
Presence of tophi at 2 years	1/517	Nurse-led education *versus* general practitioner care^24^	7/255 (2.75%) *versus* 25/262 (9.54%)	0.27 [0.11, 0.63]; N/A	Moderate^1^
			**Continuous outcomes Mean (SD): intervention** *versus* **control**	**Continuous outcomes SMD (IV, random, 95% CI);** *I*^2^%	
Change in SU, mg/dl^*^					
Outcome or subgroup	# Studies/participants	Treatment arms in theincluded studies	Mean (SD):intervention versuscontrol	SMD (IV, random, 95% CI); I2%	GRADErating
	1/1412	Pharmacist-led education and management *versus* usual care^23^	−1.67 (1.84) *versus* −1.35 (1.86)	−0.17 [−0.28, −0.07]; N/A	Moderate^1^
	1/77	Pharmacist-led education and management *versus* usual care^22^	−1.5 (0.3) *versus* 0.1 (0.3)	−5.28 [−6.25, −4.31]; N/A	Low^2^
PDC for ULT at 1 year	1/1412	Pharmacist-led education and management *versus* usual care^23^	0.66 (0.29) *versus* 0.59 (0.29)	0.24 [0.14, 0.35] N/A	Moderate^1^
Ending dose of allopurinol, mg/day^*^					
	1/1412	Pharmacist-led education and management *versus* usual care^23^	235 (104) *versus* 203 (103)	0.31 [0.20, 0.41]; N/A	Moderate^1^
	1/517	Nurse-led education *versus* general practitioner care^24^	470 (140) *versus* 240 (107)	1.85 [1.64, 2.05]; N/A	Moderate^1^
Patient satisfaction visual analogue scale (0–100 mm)	1/100	Nurse-led education *versus* no education^25^	87.5 (24.5) *versus* 75.4 (20.3)	0.53 [0.13, 0.93]; N/A	Low^2^
Patient satisfaction questionnaire (scale not reported)	1/100	Nurse-led education *versus* no education^25^	4.02 (0.4) *versus* 3.71 (0.39)	0.78 [0.37, 1.19]; N/A	Low^2^
Level of knowledge about gout (scale not reported)	1/100	Nurse-led education *versus* no education^25^	7.38 (2) *versus* 6.08 (2.24)	0.61 [0.21, 1.01]; N/A	Low^2^
Mean gout attack frequency/year during second year	1/517	Nurse-led education *versus* general practitioner care^24^	0.33 (0.93) *versus* 0.94 (2.03)	−0.38 [−0.56, −0.21]; N/A	Moderate^1^
SF-36 norm-based physical component scores	1/517	Nurse-led education *versus* general practitioner care^24^	41.31 (16.76) *versus* 37.87 (14.31)	0.22 [0.05, 0.39]; N/A	Moderate^1^

SMD is same as the effect size and is defined as SMD = (mean in experimental group)−(mean in control group)/standard deviation.

Cohen’s interpretation of effect size, which is also applicable to SMD is that 0.2, 0.5 and 0.8 are considered thresholds for a small, medium and large effect sizes respectively.

* Due to high heterogeneity in the combined analyses, results are presented and discussed separately for the two studies. GRADE evidence rating was moderate to low.

1 Level of evidence was downrated from high to moderate for the risk of bias.

2 Level of evidence was downrated from high to low for the risk of bias, and imprecision.

CI, confidence interval; GRADE, Grading of Recommendations, Assessment, Development, and Evaluation approach; IV, intravenous; M–H, Mantel-Haenszel test; N/A, not applicable; PDC, proportion of days covered; SD, standard deviation; SMD, standardized mean difference; SU, serum urate; ULT, urate-lowering therapy.

Heterogeneity as measured by I^2^ was not applicable in most instances where data were provided by only one study.

Of the three included observational studies one consisted of a nurse-delivered intervention that included education, individualized lifestyle advice and appropriate ULT use.^26^ The second study included implementing a personalized health plan (the initial interview focused on formulating a goal; the patient then selected a goal indicating their starting and desired status using a numerical scale and continuous reinforcement was achieved by weekly phone calls).^28^ The third study consisted of a pharmacist-led clinic where the patients were given information about gout and its treatment, the need for dietary and lifestyle modification and the importance of compliance with ULT.^21^

## Study quality assessment

**Figure 2.**
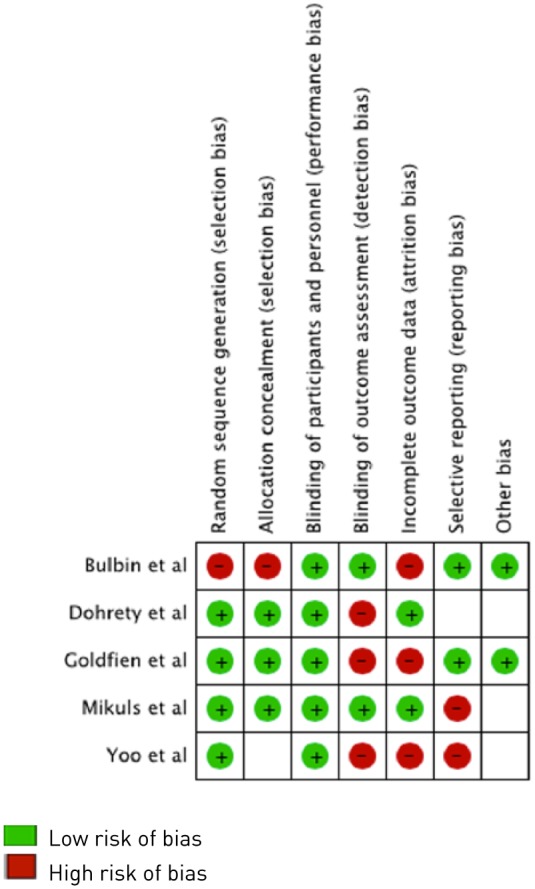
Assessment of risk of bias of the included RCTs. No color depicted means an unclear risk of bias, that is, not enough information was available to make a determination regarding that risk of bias criterion. Red indicates a high risk of bias for each criterion; green indicates a low risk of bias for each criterion. RCT, randomized controlled trial.

The risk of bias of the RCTs is presented in Figure 2. With regards to the risk of bias, although blinding of the participants and personnel was not carried out in any of the studies, most studies were deemed to be at low risk of bias. Authors of the RCTs were successfully contacted for any necessary clarifications and input regarding risk of bias of their individual studies with the exception of Yoo and colleagues^25^ where attempts were unsuccessful. The GRADE^18^ ratings are presented in Table 3. The quality score for each observational study based on the Newcastle–Ottawa scale^17^ is provided in Table 4 (range 0–9). A wide range of scores was noted.

**Table 4.** Summary of findings from the included observational studies.

Study	# patients	Type of study/quality score (range 0–9)	# sites	Findings
Rees and colleagues^26^	106	Observational cohort study/ Score 5	Multicenter	92% of participants had SU < 6 mg/dl 85% of participants had SU < 5 mg/dl Almost one-third had a reduction in number/size of the tophi at 1 year. Mean number of self-reported attacks/year reduced to 2.4 (SD 2.3)
Levya and colleagues^28^	13	Observational cohort study/ Score 4	Single center	10 of the 13 had a reduction in SU 8 of the 10 reached goal of SU ⩽ 6 mg/dl
Whiteman and colleagues^21^	52	Observational cohort study/ Score 5	Single center	73% of patients were discharged from clinic. Average SU of discharged patients (*n* = 29) decreased from 7.73 mg/dl at baseline to 4.88 mg/dl at discharge. 96.5% of discharged patients achieved a SU of 6 mg/dl. 58.66% of discharged patients achieved SU of 5.04 mg/dl. Mean percentage change in SU from baseline was 33%

SD, standard deviation; SU, serum urate. Quality score was based on the Newcastle-Ottawa scale ranging from 0-9, with a score of nine indicating the best quality for an observational study.

## Outcome measurements

Table 3 provides a summary of the effectiveness of educational/behavioral interventions for a range of outcomes in the RCTs assessed. In outcomes with one study, heterogeneity was not applicable, since this assessment requires two or more studies.

### SU < 6 mg/dl

**Figure 3.**
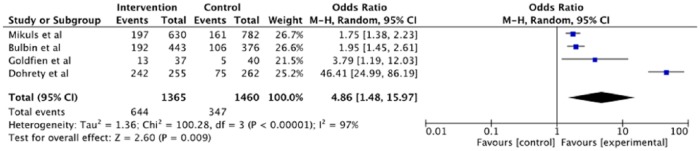
Forest plot of trials of educational/behavioral intervention *versus* no intervention (usual care) for serum urate < 6 mg/dl that shows significant benefit favoring educational/behavioral intervention, but with high heterogeneity, primarily due to a very large effect as cited in Doherty and colleagues.^24^ CI, confidence interval; M–H, Mantel-Haenszel test.

The pooled data from four RCTs^22–24,27^ with 2825 participants, found a much higher proportion of patients who underwent an educational/behavioral intervention achieved SU goal of <6 mg/dl compared with those who did not, 47.2% *versus* 23.8%, with almost five-times higher odds, with moderate quality evidence (Table 3; Figure 3).

**Figure 4.**
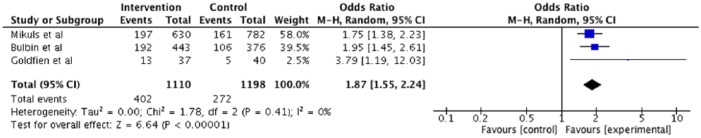
Forest plot of trials of educational/behavioral intervention *versus* no intervention (usual care) for serum urate <6 mg/dl excluding Doherty and colleagues^24^ that shows significant benefit favoring educational/behavioral intervention now with no heterogeneity. CI, confidence interval. M–H, Mantel-Haenszel test.

A sensitivity analysis excluding an outlier study with a large effect size, that is, Doherty and colleagues^24^ resulted in an OR of 1.87 (95% CI, 1.55, 2.24)) of a SU < 6 mg/dl with educational/behavioral intervention with no heterogeneity, with moderate quality evidence (Figure 4 and Table 5). Another sensitivity analysis based on using a fixed error instead of a random error was consistent with the main analyses (Table 6).

**Table 5.** Results of the main analyses followed by the sensitivity analyses with exclusion of the trial by Doherty and colleagues for the effect of the educational/behavioral intervention.^[Bibr bibr16-1759720X18807117]^

Outcome or subgroup	# studies	Intervention *versus* control	Participants	Statistical method	Effect estimate	Heterogeneity/ *I*^2^%
SU < 6 mg/dl	4	(1) Pharmacist-led education and management *versus* usual care^22^ (2) Primary care provider education *versus* usual care^27^ (3) Pharmacist-led education and management *versus* usual care^23^ (4) Nurse-led education *versus* general practitioner care^24^	2825	Odds ratio (M–H, random, 95% CI)	4.86 [1.48, 15.97]	97
SU < 6 mg/dl	3	Trial by Doherty^24^ excluded	2308	Odds ratio (M–H, random, 95% CI)	1.87 [1.55, 2.24]	0
Patients taking ULT at the end of the study period	2	(1). Primary care provider education *versus* usual care^27^ (2). Nurse-led education *versus* general practitioner care^24^	1336	Odds ratio (M–H, random, 95% CI)	5.91 [0.29, 120.97]	98
Patients taking ULT at the end of the study period	1	Trial by Doherty^24^ excluded	819	Odds ratio (M–H, random, 95% CI)	1.37 [1.04, 1.81]	N/A
Ending dose of allopurinol	2	(1). Pharmacist-led education and management *versus* usual care^23^ (2). Nurse-led education *versus* general practitioner care^24^	1929	SMD (IV, random, 95% CI)	1.08 [−0.43, 2.58]	99
Ending dose of allopurinol	1	Trial by Doherty^24^ excluded	1412	Odds ratio (M–H, random, 95% CI)	0.31 [0.20, 0.41]	N/A

CI, confidence interval; IV, intravenous; M–H, Mantel-Haenszel test; N/A, not applicable; SMD, standard mean difference; SU, serum urate; ULT, urate-lowering therapy.

**Table 6.** Results of the main and sensitivity analyses of the effect of educational/behavioral intervention using fixed vs. random error and odds vs. risk ratio.

Outcome or subgroup	# studies	Intervention *versus* control	Participants	Odds ratio (M–H, random, 95% CI); heterogeneity (I^2^%)	Odds ratio (M–H, fixed, 95% CI); heterogeneity (*I*^2^%)	Risk ratio (M–H, fixed, 95% CI); heterogeneity (*I*^2^%)	Risk ratio (M–H, random, 95% CI); heterogeneity (I^2^%)
SU < 6 mg/dl	4	Pharmacist-led education and management *versus* usual care^22^ Primary care provider education *versus* usual care^27^ Pharmacist- led education and management *versus* usual care^23^ Nurse-led education *versus* general practitioner care^24^	2825	4.86 [1.48, 15.97]; 97%	2.85 [2.42, 3.36]; 97%	1.94 [1.74, 2.16]; 93%	2.07 [1.31, 3.28]; 93%
SU < 5 mg/dl	1	Nurse-led education *versus* general practitioner care^24^	517	37.85 [22.96, 62.40]; N/A	37.85 [22.96, 62.40]; N/A	5.48 [4.14, 7.26]; N/A	5.48 [4.14, 7.26]; N/A
Presence of tophi at 2 years	1	Nurse-led education *versus* general practitioner care^24^	517	0.27 [0.11, 0.63]; N/A	0.27 [0.11, 0.63]; N/A	0.29 [0.13, 0.65]; N/A	0.29 [0.13, 0.65]; N/A
ULT adherent: PDC ⩾0.8 at 1 year	1	Pharmacist-led education and management *versus* usual care^23^	1412	1.66 [1.34, 2.05]; N/A	1.66 [1.34, 2.05]; N/A	1.34 [1.19, 1.52]; N/A	1.34 [1.19, 1.52]; N/A
Being monitored with SU at 6 months	1	Primary care provider education *versus* usual care^27^	819	3.32 [2.45, 4.51]; N/A	3.32 [2.45, 4.51]; N/A	1.48 [1.33, 1.65]; N/A	1.48 [1.33, 1.65]; N/A
Patients taking ULT at the end of the study period	2	(1). Primary care provider education *versus* usual care^27^ (2). Nurse-led education *versus* general practitioner care^24^	1336	5.91 [0.29, 120.97]; 98%	2.61 [2.06, 3.30]; 98%	1.40 [1.29, 1.52]; 97%	1.44 [0.92, 2.25]; 97%
Achieving at least a 2 mg/dl decrease in SU at week 26	1	Pharmacist-led education and management *versus* usual care^22^	77	4.26 [1.35, 13.44]; N/A	4.26 [1.35, 13.44]; N/A	3.03 [1.21, 7.58]; N/A	3.03 [1.21, 7.58]; N/A
				**SMD (IV, random, 95% CI)**	**SMD (IV, fixed, 95% CI)**	**Mean difference (IV, fixed, 95% CI)**	**Mean difference (IV, random, 95% CI)**
Patient satisfaction visual analogue scale (0–100 mm)	1	Nurse-led education *versus* no education^25^	100	0.53 [0.13, 0.93]; N/A	0.53 [0.13, 0.93]; N/A	12.10 [3.28, 20.92]; N/A	12.10 [3.28, 20.92]; N/A
Level of knowledge about gout (scale not reported)	1	Nurse-led education *versus* no education^25^	100	0.61 [0.21, 1.01]; N/A	0.61 [0.21, 1.01]; N/A	1.30 [0.47, 2.13]; N/A	1.30 [0.47, 2.13]; N/A
Patient satisfaction questionnaire (scale not reported)	1	Nurse-led education *versus* no education^25^	100	0.78 [0.37, 1.19]; N/A	0.78 [0.37, 1.19]; N/A	0.31 [0.16, 0.46]; N/A	0.31 [0.16, 0.46]; N/A
PDC at 1 year	1	Pharmacist-led education and management *versus* usual care^23^	1412	0.24 [0.14, 0.35]; N/A	0.24 [0.14, 0.35]; N/A	0.07 [0.04, 0.10]; N/A	0.07 [0.04, 0.10]; N/A
Change in SU, mg/dl	2	(1). Pharmacist-led education and management *versus* usual care^23^ (2). Pharmacist-led education and management *versus* usual care^22^	1489	−2.70 [−7.71, 2.30]; 99%	−0.23 [−0.34, −0.13]; 99%	−1.19 [−1.30, −1.08]; 99%	−0.96 [−2.22, 0.29]; 99%
Mean attack frequency during second year	1	Nurse-led education *versus* general practitioner care^24^	517	−0.38 [−0.56, −0.21]; N/A	−0.38 [−0.56, −0.21]; N/A	−0.61 [−0.88, −0.34]; N/A	−0.61 [−0.88, −0.34]; N/A
SF-36 norm-based physical component scores	1	Nurse-led education *versus* general practitioner care^24^	517	0.22 [0.05, 0.39]; N/A	0.22 [0.05, 0.39]; N/A	3.44 [0.75, 6.13]; N/A	3.44 [0.75, 6.13]; N/A
Ending dose of allopurinol	2	(1). Pharmacist-led education and management *versus* usual care^23^ (2). Nurse-led education *versus* general practitioner care^24^	1929	1.08 [−0.43, 2.58]; 99%	0.63 [0.53, 0.72]; 99%	72.22 [62.52, 81.92]; 100%	130.77 [−63.26, 324.81]; 100%

CI, confidence interval; IV, intravenous; M–H, Mantel-Haenszel test; PDC, proportion of days covered; SMD, standard mean difference; SU, serum urate; ULT, urate-lowering therapy.

N/A, not applicable, since these outcomes have only one study.

### Other SU-lowering outcomes: achieving at least a 2 mg/dl decrease in SU, SU < 5 mg/dl and a reduction in SU

Based on one RCT each, compared with usual care, those who received the educational/behavioral intervention were more likely to achieve SU < 5 mg/dl,^24^ OR was 37.85 (95% CI, 22.96, 62.40; moderate quality evidence) and achieving at least a 2 mg/dl decrease in SU at week 26,^22^ OR was 4.26 (95% CI, 1.35, 13.44; low quality evidence). Based on two RCTs^22,23^ that could not be pooled due to considerable heterogeneity (*I*^2^ = 99%), compared with usual care, the educational/behavioral intervention was associated with a greater reduction in SU with SMD 0.17 (95% CI, −0.28, −0.07; *p* = 0.001; moderate quality evidence),^23^ and SMD −5.28 (95% CI, −6.25, −4.31; (*p* < 0.00001; low quality evidence).^22^

### Medication adherence and monitoring outcomes: ULT adherence, being monitored with SU and taking ULT at the end of the study period and the ending dose of allopurinol

Overall, one RCT reported allopurinol adherence based on the proportion of days covered (PDC) at 1 year, that is, allopurinol prescription refills of ⩾0.80^23^ and the likelihood of SU being monitored at 6 months.^23^ The use of the educational/behavioral intervention was associated with a significantly higher proportion with allopurinol PDC ⩾ 0.80, OR 1.66 (95% CI, 1.34, 2.05) and being monitored with SU at 6 months OR 3.32 (95% CI, 2.45, 4.51). The evidence was moderate quality for both.

Based on two RCTs^24,27^ of ULT continuation at the end of the study period at 6 months^27^ (or 2 years^24^) that could not be pooled due to considerable heterogeneity (*I*^2^ = 98%), compared with usual care (primary care provider education^24^) and nurse-led education^27^ were each associated with significantly higher odds of ULT continuation at 6 and 24 months, 26.50 (95% CI 12.58, 55.80) and 1.37 (95% CI 1.04, 1.81), respectively. The evidence was moderate quality for both studies.

Based on two RCTs with moderate quality evidence,^23,24^ reporting on the ending dose of allopurinol that could not be pooled due to considerable heterogeneity (*I*^2^ = 99%), compared with usual care, the nurse-led^24^ or the pharmacist led^23^ educational/behavioral intervention were each associated with a higher ending dose of allopurinol, respective SMDs were 1.85 (95% CI, 1.64, 2.05; *p* < 0.00001) and 0.31 (95% CI, 0.20, 0.41; *p* < 0.00001). The evidence was moderate quality for both studies.

### Presence of tophi at 2 years and the mean gout attack/flare frequency

Based on one RCT with moderate quality evidence, compared with usual care, those who received the educational/behavioral intervention, had a lower likelihood of presence of tophi at 2 years. The OR was 0.27 (95% CI, 0.11, 0.63)^24^ and lower mean gout attack frequency, SMD was −0.38 (95% CI, −0.56, −0.21; *p* < 0.0001).^24^

### Level of knowledge about gout, patient satisfaction and quality of life

Based on one RCT each, with low quality evidence, compared with usual care, the educational/behavioral intervention in gout was associated with higher patient knowledge about gout,^25^ patient satisfaction^25^ and SF-36 norm-based physical component scores at 2 years,^24^ the respective SMDs were 0.61 (95% CI, 0.21, 1.01; *p* = 0.003), 0.78 (95% CI, 0.37, 1.19; *p* = 0.0002)) and 0.22 (95% CI, 0.05, 0.39; *p* = 0.01).

### Observational studies assessing educational and behavioral interventions for a range of outcomes

There were three observational studies that met inclusion criteria. Rees and colleagues^26^ tested the effectiveness of a nurse-delivered intervention that included education, individualized lifestyle advice and appropriate ULT. Their goal was to achieve a SU ⩽ 360 μmol/l (equivalent to SU ⩽ 6 mg/dl) at 1 year. Following the intervention, 92% of participants had SU < 360 μmol/l and 85% of participants had SU < 300 μmol/l. In the 17 patients with tophi at baseline almost one-third had a reduction in the number or size of the tophi at 1 year. The mean number of self-reported attacks/year reduced from 4 (SD 4) to 2.4 (SD 2.3) following the nurse-delivered intervention.

Levya and colleagues^28^ formulated a gout personalized health plan in 13 people with gout. Patients selected a goal congruent with gout management, such as improving diet, stopping alcohol consumption or increasing physical activity, indicating their starting and desired status using a numerical scale. Continuous reinforcement was achieved by weekly physician phone calls. SU was measured at baseline and 3 months, that is, the end of the study. A total of 10 of the 13 people had a reduction in SU and 8 of the 10 reached the goal of SU ⩽ 6 mg/dl.

Whiteman and colleagues^21^ evaluated the effectiveness of a monthly pharmacist-led gout clinic where patients were given information about gout and its treatment, the need for dietary and lifestyle modification and the importance of compliance with ULT. Patients were offered ongoing clinical review and monitoring/adjustment of treatment until their SU was within target range after which they were discharged back to their primary care. Overall, 73% of patients were discharged from the clinic. The average SU of discharged patients decreased from 460 μmol/l at baseline to 290 μmol/l at discharge. A total of 96.5% of discharged patients achieved SU of 360 μmol/l. Overall, 58.6% of discharged patients achieved SU of 300 μmol/l. The mean percentage change in SU from baseline was 33%.

## Discussion

Both the ACR and the EULAR treatment guidelines regard patient education in gout an overarching principle of gout therapy.^2,29^ To our knowledge, this is the first systematic review to examine the effectiveness of educational or behavioral interventions to improve outcomes in gout. We demonstrated that educational and behavioral healthcare interventions are effective in improving one or more clinically important outcome and patient-reported outcomes in people with gout at short to intermediate follow up, based on moderate to low quality evidence from trials and observational studies. Nurse-led interventions, pharmacist-led programs and a physician/multidisciplinary approach, all of which included patient or physician education as a key component, were more effective than the comparator, that is, usual care in achieving target SU. This is not surprising, since gout knowledge and treatment gaps have been well highlighted in the literature^30–39^ including limited health literacy.^40^ Harrold and colleagues^41^ showed that knowledge deficits about dietary triggers and chronic medications were common in patients with gout and worse in those with active gout. Many in the general public associate gout with negative stereotypes and trivialize of the impact of disease despite its severity.^33^

Nonadherence compromises long-term treatment effectiveness in patients with gout and is a substantial roadblock to achieving better outcomes. Adherence with ULT can reduce gout flares,^42,43^ tophus size,^42,43^ improve quality of life,^42,43^ activity limitation^42,43^ and survival.^44,45^ Improved gout management can reduce the substantial economic burden associated with uncontrolled gout.^46^ Gout patients with poorly controlled SU have higher healthcare costs than patients whose SU are better controlled.^47,48^ In view of this, a cost-effective means of maintaining ULT adherence, and thereby reducing the prevalence of gouty flares, is highly desirable.

Currently gout management is suboptimal despite excellent available therapy, most of which is affordable. Educational and behavioral interventions focusing on gout self-management including ULT adherence may constitute the cornerstone of gout management. The multifaceted interventions should provide patient education on gout and its causes, effect of diet and exercise and pharmacotherapy. Education and counseling aims to change the negative thoughts about medications and increase motivation.^49^ Engagement of clinic staff, onsite live and enduring online education and surveys of provider performance improvement preferences were also effective in achieving target SU and ULT use. Therefore, findings from our systematic review further emphasize the crucial role of education of the patients as well as providers can play in improving gout outcomes. Even though interventions can be broadly categorized as having a predominant behavioral or educational component, we acknowledge that the educational and behavioral components overlap considerably in some intervention strategies.

It is difficult to apply principles from RCTs of therapeutic agents where blinding is possible and appropriate in optimal design to education/behavioral interventions where blinding of patients in the treatment arm is usually not possible. There is a paucity of strategies to mitigate this. The risk of bias tool was adapted, bearing in mind that the nature of intervention under review precludes blinding of participants. Behavioral or educational interventions for gout are low-risk interventions *versus* pharmacologic option for gout, since they have few or no unanticipated harms. Therefore, even a small effect size for an education/behavioral intervention can lead to its implementation since the downside of its implementation are usually limited, that is, up-front cost and rarely unanticipated consequences.

We used the GRADE^18^ approach to rate the quality of the evidence and reflect the extent to which we are confident that the effect estimates are correct. This was done to improve clarity and make judgments more transparent. For example, a moderate effect, that is, SMD of 0.5 or more, with moderate to high quality evidence reflects high confidence that the estimate is unlikely to change with more research studies. On the other hand, a small effect (SMD of 0.2 or lower) with low quality evidence is something which is likely to change with more research and may not indicate a meaningful difference with the intervention.

There are several limitations of our review. First, data for several disease outcomes came from a small number of trials. Second, health literacy levels and socioeconomic status were not reported in most trials, and these can affect outcomes, and help us better understand why and in whom certain educational interventions will and will not work. Many patients do not understand disease-related information provided in written and verbal form in rheumatology medical encounters.^50^ It was also unclear from some studies whether the educational interventions were ongoing or spaced whereby the patient is presented with the educational concept or learning objective, a period of time is allowed to pass and then they are presented the same concept again repeatedly over intervals of time. Research has shown that spaced learning is more efficient in comparison with standard teaching and leads to improved educational practices.^51^

We identified several knowledge gaps in this field of research. We concluded that the current evidence for gout educational/behavioral interventions failed to provide a perspective on the long-term sustainability of the intervention, transferability of effective interventions to a different setting or assess the impact on long-term gout outcomes. We found that all of the studies of gout educational/behavioral interventions delivered education either *via* written or verbal education modalities. None involved a technology-based intervention for gout. There are a number of freely available online patient information resources for patients with gout,^52^ however their effectiveness to improve patient outcomes has not been explored systematically, and the quality of information varies widely between resources. How and what role these online patient information resources can play in the improvement of gout outcomes is currently unknown. It is paramount that socioeconomic factors, health literacy and educational level be taken into account since they can affect ability to access programs.^53^ Interventions should ideally emphasize issues, which motivate patients to adhere to treatment based on their priorities and address identified barriers to self-management. No cost information was provided in these studies. We anticipate that the cost for the development and implementation of these educational or behavioral interventions (including materials, electronic technology interventions or delivery) will likely be lower than the cost of unwanted high-cost health care utilization in urgent care, emergency room and inpatient settings resulting from the treatment of uncontrolled gout (gout flares, or gout flares complicating acute medical problems or surgical procedures). We suspect that educational and behavioral interventions in gout are likely cost-effective, but also recommend that cost-reporting methods should be reported in future studies to allow a complete analysis of cost and cost-effectiveness of educational and behavioral interventions in gout.

In summary, multifaceted gout educational/behavioral interventions were successful in improving both ULT adherence and clinical outcomes in gout in the short to intermediate term. These interventions involved the ancillary staff, pharmacist or the physician. Implementing these interventions in one’s practice is feasible and could lead to improved patient outcomes in gout. Given the nature of gout as a chronic illness, ongoing supportive services may be necessary especially at critical points of treatment. It seems necessary to tailor interventions to the speciﬁc clinical situation (acute flare *versus* chronic gout treatment) as well as different treatment settings (primary *versus* inpatient). Given the importance of improved ULT adherence and gout self-management, more rigorous and well-conducted studies are needed. Educational/behavioral interventions can improve adherence and persistence with gout treatments. Different approaches may be needed based on age, sex, educational level, health literacy, ethnicity, language, and other factors which affect ability to access programs.^53^ There is currently a lack of studies in several gout subpopulations including women, racial/ethnic minorities, the elderly, and people with limited health and graphical literacy and numeracy. Future research seeking to improve gout adherence should take into account both the compliance and persistence aspects of adherence.

## Conclusion

In this systematic review and meta-analysis of RCTs and observational studies, multifaceted gout educational/behavioral interventions were associated with achieving a goal SU < 6 mg/dl and a reduction in the SU level, adherence to ULT, higher ULT adherence and higher ULT dose, SU monitoring, reduction in the number of tophi, patient satisfaction, improved patient knowledge about gout, lower gout attack frequency and better physical health status, with moderate to low quality evidence. These findings support further exploration of educational and behavioral interventions in patients with gout, and their potential use in improving gout outcomes in clinical settings in the near future. Several opportunities for testing multifaceted gout educational/behavioral interventions are also identified.
